# Effect of cereal soaking and carbohydrase supplementation on growth, nutrient digestibility and intestinal microbiota in liquid-fed grow-finishing pigs

**DOI:** 10.1038/s41598-020-57668-6

**Published:** 2020-01-23

**Authors:** Alberto Torres-Pitarch, Gillian E. Gardiner, Paul Cormican, Mary Rea, Fiona Crispie, John V. O’Doherty, Pierre Cozannet, Tomas Ryan, Peadar G. Lawlor

**Affiliations:** 1 Teagasc, Pig Development Department, Animal and Grassland Research and Innovation Centre, Moorepark, Fermoy, County Cork Ireland; 2 School of Agriculture and Food Science, University College Dublin, Belfield Dublin, Ireland; 3 Department of Science, Waterford Institute of Technology, Waterford, Ireland; 4 Animal and Bioscience Research Department, Animal and Grassland Research and Innovation Centre, Teagasc, Grange, County Meath Ireland; 5 Teagasc Food Research Centre, Moorepark, Fermoy, County Cork Ireland; 6 APC Microbiome Ireland, University College Cork, Cork, Ireland; 7 Adisseo France SAS, Antony, France

**Keywords:** Applied microbiology, Microbiome

## Abstract

Soaking the cereal fraction of a liquid diet prior to feeding (C_soak_), and/or carbohydrase enzyme supplementation (ENZ) are likely to modulate both feed and intestinal microbial populations and improve feed efficiency (FE) in pigs. To test this hypothesis, a total of 392 grow-finisher pigs (~33.4 kg, 7 pigs/pen) were randomly allocated to 4 treatments in a 2 × 2 factorial arrangement for 70 days as follows: (1) fresh liquid feed (Fresh); (2) Cereal soaked liquid feed (Soak); (3) Fresh + ENZ and (4) Soak + ENZ. An interaction between ENZ and C_soak_ was found for average daily gain (ADG) during the growing phase (day 0 to 21; P < 0.05) where pigs fed the Soak + ENZ diet had higher ADG than pigs fed the Fresh + ENZ diet. No treatment effect was found for ADG thereafter. Enzyme supplementation increased total tract nutrient digestibility (P < 0.05) and reduced caecal VFA concentrations (P < 0.05) but did not improve pig growth or FE. Both C_soak_ and ENZ modulated intestinal microbiota composition; increasing abundance of bacterial taxa that were negatively correlated with pig growth and reducing abundance of taxa positively correlated with pig growth and caecal butyrate concentration. In conclusion, both strategies (C_soak_ and ENZ) improved nutrient digestibility in pigs and modulated intestinal microbiota composition.

## Introduction

Nutritional strategies to improve feed efficiency are of particular interest as their use will reduce the environmental impact and improve profitability of pig production^1,2^. The intestinal microbiota plays an important role in the digestion process and can impact feed efficiency in pigs^3–5^. The type of carbohydrate substrates present in the intestine influence intestinal microbiota composition^6–8^.

The pig’s digestive system lacks specific enzymes to break down some of the chemical bonds present in the non-starch polysaccharide (NSP) fraction of the diet (i.e. arabinoxylans and β-glucans). Dietary carbohydrase supplementation is therefore used as a strategy to improve nutrient digestibility, increase growth and improve feed efficiency^9–11^. Xylanase and β-glucanase (XB) are carbohydrases that can break down arabinoxylans and β-glucans into monomers of xylose, arabinose and glucans. These substrates may be used as a direct source of energy for pigs via absorption from the small intestine but they can also undergo microbial fermentation, producing volatile fatty acids (VFA), which when absorbed from the intestine also contribute to the pig’s energy supply^12^. Dietary XB supplementation can influence the abundance of selected enteric bacterial groups (e.g. *Bifidobacterium* spp. and *Lactobacillus* spp.) in pigs^13–16^. However, sequence-based microbial profiling of enzyme-supplemented pigs has not been conducted to date and the microbial species that most efficiently utilise xylose, arabinose and glucans are as yet unknown. A large number of publications have examined the effects of dietary XB supplementation on the feed efficiency of grow-finisher pigs^9–11,16–20^. Despite consistent increases in nutrient digestibility, improvements in growth or feed efficiency are not always observed when XB is supplemented to pig diets^21^. Examining the effect of XB dietary supplementation on intestinal bacterial composition may help to explain the variability in feed efficiency response to XB supplementation.

Endogenous NSP-degrading enzymes are also naturally present in plant ingredients used in pig diets^22,23^. These endogenous enzymes are activated when they come into contact with water and therefore, soaking feed prior to feeding has been suggested as a strategy to increase nutrient digestibility in pigs^24–26^. However, liquid feeding may have negative consequences, such as increased bacterial degradation of free essential amino acids (AA) during preparation and feeding and this requires particular consideration when using such systems^27,28^. Soaking only the cereal fraction of liquid diets prior to feeding (C_soak_) may be a useful strategy to improve the microbial quality and nutritional value of pig feed without reducing it’s free AA content, the latter being supplied in the balancer fraction (containing soybean meal, synthetic AA, minerals and vitamins) which is  only incorporated into the diet immediately before feeding.

Both strategies, namely C_soak_ and supplementation with a carbohydrase enzyme complex (ENZ), may release substrates for use by microbes present in the liquid feed as well as by those in the gastrointestinal tract (GIT) of the pig. It was therefore hypothesized that soaking the cereal component of the diet with or without ENZ supplementation would modulate the microbiota in the feed, thereby favourably impacting the intestinal microbiota and as a consequence improve growth and feed efficiency of grow-finishing pigs. The objective of this study was to determine the impact of both strategies on the feed and intestinal bacterial composition, nutrient digestibility, pig growth, feed efficiency and intestinal VFA production. Correlations between growth parameters, intestinal VFA concentrations and the relative abundance of microbial taxa found to be differentially abundant due to treatment (C_soak_ and ENZ) were conducted to find associations between growth parameters and microbial activity.

## Material and Methods

### Experimental design

A total of 392 pigs [Maxgrow x (Landrace x Large White); Hermitage Genetics, Sion Road, Kilkenny, Ireland] with an initial live weight (LW) of 33.4 (±0.88 SEM) kg were penned in groups of 7 pigs of the same sex per pen. The pigs were blocked on the basis of sex (female and entire male) and initial body weight and assigned to dietary treatments in a randomised block design. The experiment was conducted in two batches; the first had 9 replicated pens (5 male and 4 female) and the second run had 5 replicated pens (3 male and 2 female) per dietary treatment. The duration of the experiment was 71 days and the experiment was arranged as a 2 × 2 factorial. The factors were: soaking of the dietary cereal fraction (C_soak_; un-soaked *vs*. soaked) for 3 h prior to mixing with balancer (containing soybean meal, synthetic AA, minerals and vitamins) followed by immediate feeding and supplementation of cereals with XB [Enzyme (ENZ); no vs. yes]. The dietary treatments were: (1) fresh liquid feed (Fresh); (2) C_soak_ liquid feed (Soak); (3) Fresh + ENZ and (4) Soak + ENZ. All diets were formulated to contain 9.4 MJ NE/kg [7% below the NRC requirements^29^] and 9.15 g/kg of standardised ileal digestible lysine [SID; at the NRC requirements^29^] to avoid the potential impact of liquid feeding on AA degradation (Table 1). All other AA were formulated relative to lysine according to the ideal protein concept^29^. The XB enzyme (Rovabio® Excel AP, Adisseo France SAS, Antony, France) was derived from *Talaromyces versatilis* sp. and provided 22,000 viscosity units (VU) of endo-1,4-β-xylanase (EC 3.2.1.8) and 30,000 VU of endo-1,3(4)-β-glucanase (EC 3.2.1.6) per gram of product. The enzyme was supplemented to the cereal fraction of the diet prior to soaking at 120 g/tonne of cereal mix in order to provide 100 g/tonne of finished feed [2200 VU of Xylanase and 3000 VU of β-glucanase per kg of finished diet, 88% dry matter (DM) basis].

**Table 1:** Ingredient and nutrient composition of dietary components and experimental diets ^a^(on an air dry basis, g/kg unless otherwise stated).

	Dietary components			Experimental diets	
	CER	CER + ENZ	BAL	Basal	Basal + ENZ
**Ingredient composition**^b^					
Barley	450.60	450.48	0.00	377.30	377.19
Wheat	418.00	418.00	0.00	350.00	349.99
Soybean meal	0.00	0.00	829.60	135.00	134.97
Wheat feed	131.40	131.40	0.00	110.00	110.02
Limestone	0.00	0.00	76.80	12.50	12.50
Lysine HCl	0.00	0.00	26.90	4.40	4.37
Mono dicalcium phosphate	0.00	0.00	22.60	3.70	3.68
Salt	0.00	0.00	18.40	3.00	3.00
L-Threonine	0.00	0.00	6.70	1.10	1.09
Soya oil	0.00	0.00	6.10	1.00	1.00
Vitamin and mineral premix^c^	0.00	0.00	6.10	1.00	1.00
DL-Methionine	0.00	0.00	4.90	0.80	0.80
Celite	0.00	0.00	1.80	0.30	0.30
Enzyme^d^	0.00	0.12	0.00	0.00	0.10
**Component composition**^b^					
Cereal fraction	1000.00	1000.00	0.00	837.30	837.30
Balancer fraction	0.00	0.00	1000.00	162.70	162.70
**Nutrient composition**^**e**^					
Dry matter	878.0	879.0	903.0	882.1	882.9
Crude protein	103.0	106.0	409.0	153.2	153.2
Ash	22.0	22.0	173.6	46.6	46.6
Oil	27.0	26.5	26.3	26.8	26.5
Crude fibre	47.0	46.0	25.0	42.5	42.5
Neutral detergent fibre	157.0	152.0	70.0	142.8	138.7
Acid detergent fibre	57.9	57.4	36.7	54.5	54.0
Net energy, MJ/kg	—	—	—	9.40	9.40
Total lysine	3.4	3.4	41.0	9.52	9.52
SID^f^ lysine,	—	—	—	9.15	9.15
Total Ca,	—	—	—	6.48	6.48
Digestible P	—	—	—	2.40	2.40
Arabinoxylans	—	—	—	86.88	86.88
β-glucans	—	—	—	22.82	22.82
Xylanase activity^g^, VU/kg	0	5748	—	0	4770
β-glucanase activity^g^, VU/kg	0	6439	—	0	5344

^a^CER = cereal fraction of the diet, CER + ENZ = cereal fraction of the diet supplemented with a carbohydrase enzyme (xylanase and β-glucanase, XB), BAL = balancer fraction (non-cereal component) of the diet.

^b^Calculated values.

^c^Vitamin and mineral premix provided per kilogram of complete diet (on an air basis): Cu from copper sulphate, 15 mg; Fe from ferrous sulphate monohydrate, 24 mg; Mn from manganese oxide, 31 mg; Zn from zinc oxide, 80 mg; I from potassium iodate, 0.3 mg; Se from sodium selenite, 0.2 mg; retinyl acetate 0.7 mg; cholecalciferol, 12.5 μg; DL-alpha-tocopheryl acetate, 40 mg; Vitamin K, 4 mg; vitamin B12, 15 μg; riboflavin, 2 mg; nicotinic acid, 12 mg; pantothenic acid, 10 mg; vitamin B1, 2 mg; vitamin B6, 3 mg; and celite, 300 mg.

^d^Carbohydrase complex based on xylanase and β-glucanase (Rovabio® Excel AP, Adisseo France SAS, Antony, France) providing a minimum guaranteed content of 2200 VU and 3000 VU, respectively, per kg of finished diet (on an air dry basis).

^e^Analysed values for dietary components (CER, CER + ENZ and BAL). For the experimental diets (basal and basal + ENZ) values given are calculated from the analysed dietary component values. Values with a “-” were not analysed and the calculated values given for the experimental diets are from the calculated values in the matrix formulation.

^f^SID = Standardized ileal digestibility.

^g^One viscosity unit (VU) is defined as the amount of enzyme reducing the viscosity of the solution, to give a change in relative fluidity of 1 dimensionless unit per minute per g at pH 5.5 and 30 °C.

### Feed preparation and animal management

Three dietary components were manufactured in meal form at the Teagasc feed mill facilities (Teagasc, Moorepark, Fermoy, Co. Cork, Ireland): (1) Cereal fraction (CER) composed of a mixture of barley (45%), wheat (42%) and wheat feed (12%) which were ground through a 3 mm screen before mixing; (2) CER supplemented with the XB enzyme complex (CER + ENZ) and (3) Balancer fraction (BAL) consisting of a mixture of soya bean meal, synthetic AA, vitamins and minerals. The three dietary components were transported to the adjoining experimental farm and stored in steel bins during the experimental period. The liquid dietary treatments were prepared and provided to the pigs at the experimental farm. The ingredient and nutrient composition of the dietary components and the basal diets are reported in Table 1. The computerised liquid feeding system (HydroMix, Big Dutchman, Germany) consisted of two mixing tanks (500 L), each equipped with an agitator (consisting of 1 vertical axis and 6 horizontal blades) and a high-pressure air system for delivery of the feed from the mixing tanks to the pen troughs, each of which was fitted with a solenoid valve and an electronic feed sensor. The electronic sensors were checked 6 times a day and troughs with feed below the level of the sensor were refilled with their respective dietary treatments. To prepare the fresh liquid dietary treatments (Fresh and Fresh + ENZ), the CER (or CER + ENZ) and BAL at the correct ratio (0.837:0.163, CER:BAL) were mixed with water in a 1: 2.5 ratio (fresh feed: water; 25.1 DM%) and agitated for 5 min before delivery to troughs. To prepare the C_soak_ dietary treatments (Soak and Soak + ENZ) the CER (or CER + XB) was mixed with water (25.1 DM%) and agitated for 3 h prior to mixing with BAL and water to achieve a 25.1 DM% mixture which was then agitated for 5 min, after which it was pumped to the troughs.

The groups of 7 pigs were penned in slatted pens (2.37 m × 2.36 m) with solid PVC partitions. The feeders were short stainless steel troughs (100 cm × 32.5 cm × 21 cm) located on top of a rubber mat (1.5 × 1 m) to help minimise feed wastage. Each pen was provided with a drinking bowl (DRIK-O-MAT, Egebjerg International A/.S, Egebjerg, Denmark). Air temperature was maintained at 20 to 22 °C. Pigs were observed closely twice daily. Any pig showing signs of ill-health was treated as appropriate. All veterinary treatments were recorded including identity of pig, symptom, medication used and dosage.

### Recordings and sample collection

Individual pig LW and feed disappearance per pen were recorded on days 0, 21 and 70 of the experiment, from which ADG, ADFI and FCR were calculated. At days 2 and 15 of the experiment, ~600 g of liquid cereal was collected from the mixing tanks immediately after water addition (0 h) and after soaking for 3 h. In addition, complete liquid diet samples for each dietary treatment were collected from the mixing tank and from the pen troughs on both days. Samples from the pen troughs were collected 30 min before a new feed mix was dispensed into the trough. Three 1.5 ml aliquots of each sample were immediately snap-frozen in liquid nitrogen and stored at −80 °C for subsequent microbiota analysis. Another aliquot of the sample (~20 mL) was frozen and stored at −20 °C for subsequent VFA analysis. The rest of the sample was frozen at −20 °C in aluminium foil trays for subsequent freeze-drying prior to chemical analysis. Fresh faecal samples from 6 pens (pooled from 3 pigs/pen) per treatment were collected daily for two days prior to slaughter and the corresponding feed for each pen was collected 1 day before faecal collection. Feed and faecal samples were stored at −20 °C for subsequent apparent total tract digestibility (ATTD) determination. At day 70 of the experimental period, pigs were transported to a commercial abattoir (Dawn Pork and Bacon, Waterford, Ireland), stunned using CO_2_ and killed by exsanguination. At the slaughter of the first run (36 pens, 9 replicates), the intestinal tracts of 22 pigs per treatment (2 and 3 pigs per pen of males and females, respectively) were recovered. Digesta samples were collected from the terminal ileum (1.5 m proximal to the ileo-caecal valve) and the blind end of the caecum. Three aliquots of ileal digesta were stored as for the feed samples: one aliquot (~5 mL) for microbiota analysis, a second aliquot (~20 mL) for VFA analysis and the remainder for apparent ileal digestibility (AiD) determination. Two aliquots of caecal digesta were stored; one for microbiota analysis (~5 mL; snap-frozen and stored at −80 °C) and a second for VFA analysis (~20 mL; stored at −20 °C). Hot carcass weight was recorded 45 min after stunning, and back-fat thickness and muscle depth, measured at 6 cm from the edge of the split back at the level of the 3rd and 4th last rib, were determined using a Hennessy Grading Probe (Hennessy and Chong, Auckland, New Zealand). Lean meat content was estimated according to the following formula: Estimated lean meat content (%) = 60.3–0.847x + 0.147y where x = fat depth (mm); y = muscle depth (mm) (Department of Agriculture Food and Rural Development, 2001).

### Feed analysis

The dietary components (CER, CER + XB, and BAL) were ground through a 1 mm screen in a Cyclotec^TM^ mill (FOSS electric, Hilleroed, Denmark) and analysed for DM, ash, fat, gross energy (GE), crude fibre (CF), neutral detergent fibre (NDF), acid detergent fibre (ADF) and crude protein (CP) as described by Clarke *et al*.^12^ and Torres-Pitarch *et al*.^30^. Amino acid concentrations were determined using high performance liquid chromatography^31^. The liquid feed samples collected from the mixing tanks and the pen troughs were freeze-dried prior to grinding through a 1 mm screen and analysed as outlined above, as well as for biogenic amines. Biogenic amines were analysed by Sciantec Ltd. (United Kingdom) by extraction with 10% trichoroacetic acid solution and subsequent ion exchange chromatography. The cereal dietary components (CER and CER + XB) were analysed by ADISSEO France for xylanase activity using a colorimetric assay. Values for the complete diets were calculated from the analysed values of each dietary component (Table 1). One VU of endo-1,4-β-xylanase activity was defined as the amount of enzyme reducing the viscosity of the solution, to give a change in relative fluidity of 1 dimensionless unit per minute per mL (or per g) under the conditions of the assay (pH 5.5 and 30 °C).

### Nutrient digestibility analysis

The freeze-dried feed, faeces and ileal digesta samples collected for digestibility determination were individually ground through a 1 mm screen using the Cyclotec^TM^ mill. After milling, each sample type was pooled by pen (n = 9 per treatment) and analysed for DM, ash, acid insoluble ash (AIA), GE and CP for determination of AiD and ATTD. The concentration of AIA was determined according to the method of McCarthy *et al*.^32^.

### Volatile fatty acid analysis and pH of feed and ileal and caecal digesta samples

Feed and ileal and caecal digesta samples were thawed to room temperature and pH was measured using a pH meter (F2-Meter, Mettler Toledo, Germany). Volatile fatty acid concentrations were analysed in duplicate for liquid CER and liquid feed, as well as ileal and caecal digesta samples using gas liquid chromatography according to the method described by Clarke *et al*.^12^ but instead of ~1 g of initial sample ~3.5 g was used for the extraction.

### Microbial analysis of liquid feed, ileal and caecal digesta samples

Feed samples collected from the mixing tank and from the pen troughs (10 g) were homogenized in 90 ml of maximum recovery diluent (MRD) and a 10-fold dilution series was performed in MRD. Appropriate dilutions were plated in duplicate as follows; (1) pour-plated on De Man, Rogosa and Sharpe (MRS) agar containing 50 U/mL nystatin (Sigma-Aldrich, Arklow, Co. Wicklow, Ireland), overlaid and incubated at 30 °C for 72 h for lactic acid bacteria (LAB); (2) pour-plated on Violet Red Bile Dextrose (VRBD) agar, overlaid and incubated at 37 °C for 24 h for *Enterobacteriaceae*; and (3) spread-plated on Yeast Glucose Chloramphenicol (YGC) agar incubated at 25 °C for 5 days for yeasts and moulds. Colonies were counted and the counts averaged and presented as CFU/g of the original sample. All microbiological media were obtained from Merck (Darmstadt, Germany). Total DNA was extracted from the liquid feed, ileal and caecal samples using the QIAamp DNA stool minikit (Qiagen, Crawley, United Kingdom) according to the manufacturer’s instructions, apart from adding a bead beating step after sample addition to the InhibitEX buffer and increasing the lysis temperature to 95 °C to increase the DNA yield^33^. Microbial profiling was performed using high-throughput sequencing of the V3-V4 region of the 16S rRNA gene (paired end reads of 250 bp) on an Illumina MiSeq platform according to the standard Illumina protocol, except that the PCR mix volume was doubled in the first PCR step and 30 cycles were used instead of 25^34^. Pair-end reads in all samples were quality assessed using FastQC v0.11.7. BBduk from the BBTools suite (https://jgi.doe.gov/data-and-tools/bbtools/) was used to quality trim (cuttoff- phred = 20). Primers and low quality read tails were also removed. The DADA2 pipeline (v1.6) was used to perform read filtering and dereplication, chimera detection and removal, read-pair merging and inference of amplicon sequence variants (ASV) in each sample. Taxonomy was assigned to each derived ASV using a naive Bayesian classifier method against the Silva database (Version 128). Species level was identified, when possible, by blasting the sequences against the nucleotide database of the U.S. National Center for Biotechnology Information (NCBI). Alpha diversity indices (Chao1, Shannon and Simpson) based on subsampled read data (n = 32,500 reads per sample) and β-diversity (Bray-Curtis) analyses were calculated using the phyloseq R Package^35^. Data were subsequently plotted using the ggplot2 R package^35^.

### Statistical analysis

Growth parameters (LW, ADG, ADFI and FCR), carcass quality parameters, nutrient digestibility, digesta pH and VFA concentrations were analysed using the PROC MIXED procedure of SAS® software version 9.4 (SAS Institute, Inc., Cary, NC, US). For growth parameters; C_soak_, ENZ supplementation, time, sex and their associated interactions were included in the model as fixed effects while initial LW was included as a covariate in the model and day was regarded as a repeated variable with pen as the experimental unit. For carcass quality parameters, nutrient digestibility, digesta pH and VFA concentrations; C_soak_, ENZ supplementation, sex and their associated interactions were included in the model as fixed effects with pen as the experimental unit; for kill out percentage, muscle depth, fat depth and lean meat percentage; carcass weight was included as a covariate in the model. A compound symmetry covariance structure was fitted to all data. Model suitability was investigated by checking normality of scaled residuals using the Shapiro-Wilk test within the UNIVARIATE procedure of SAS. The results were presented as least square means ± SEM. Microbial relative abundance at phylum, family, genus and ASV levels were analysed using generalized linear mixed model equation methods in the PROC GLIMMIX procedure of SAS®. Each taxon was compared in a univariate manner and the p-values were corrected for multiple comparisons using a Benjamini-Hochberg estimated false discovery rate (FDR). A gamma distribution was assumed for all data. Models included C_soak_, ENZ supplementation and their interaction as fixed effects. In all models, data were back transformed to the original distribution using the ilink option. Spearman correlations between the differentially abundant genera and ADG, carcass quality data, digesta pH and digesta VFA concentrations were determined using base R^35^ using the individual pig as the experimental unit. Significance was reported for P ≤ 0.05.

### Ethics approval

Ethical approval for this study was granted by the Teagasc Animal Ethics Committee (approval no. TAEC86/2015). The experiment was conducted in accordance with Irish legislation (SI no. 543/2012) and the EU Directive 2010/63/EU for animal experimentation.

## Results

### Characterization of dietary treatments sampled before mixing, from the mixing tank and from the troughs

**Figure 1:**
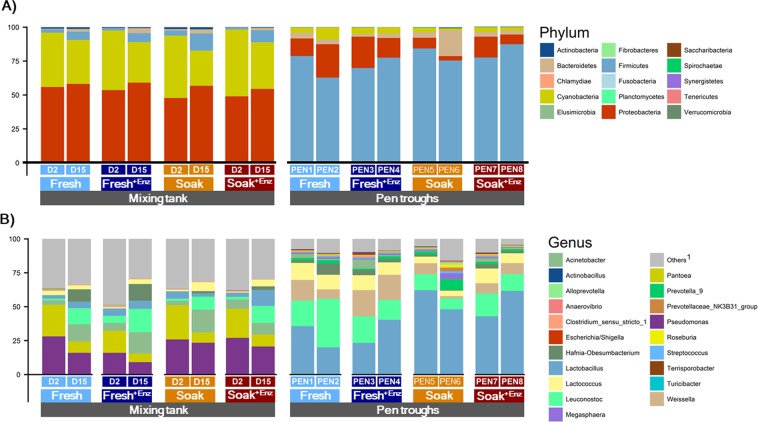
Relative abundance (%) of bacterial phyla (**A**) and the 20 most abundant bacterial genera (**B**) present in the dietary treatments in the mixing tanks (collected at day 2 or day 15 of the experiment) and in the feed troughs in the pig pens. The dietary treatments were: Fresh = liquid feed prepared with fresh ingredients (); Fresh + ENZ = Liquid fresh feed supplemented with a xylanase and β-glucanase (XB) carbohydrase enzyme (); Soak = Liquid feed prepared with 3h-soaked cereals (); Soak + ENZ = Liquid feed prepared with 3h-soaked cereals supplemented with the XB enzyme (). ^1^Others = All bacterial genera not included in the 20 most abundant.

The calculated and analysed composition of the dietary components and the basal diets are presented in Table 1. The analysed values for GE, CF, CP and lysine were as expected. The pH, VFA concentrations, and selected microbial counts of the dietary treatments collected from the mixing tanks and the pen troughs are presented in Table 2. A lower pH (5.4 vs. 6.2, SD 0.03, n = 2), higher total VFA concentration (30.6 *vs*. 15.4 mmol/g, SD 1.39, n = 2; mainly composed of acetate), higher LAB counts (8.9 vs. 6.3 log_10_ CFU/g, SD 1.52, n = 2), and higher yeast counts (6.2 vs. 5.2 log_10_ CFU/g, n = 2) were observed in samples collected from pen troughs compared to those collected from the mixing tanks. The analysed nutrient composition and biogenic amine concentrations of the dietary treatments collected from the feed troughs are reported in Table 3. No major differences in nutrient composition were observed among dietary treatments. The average concentration of biogenic amines found in the feed troughs was 75, 14 and 9 ppm for cadaverine, spermidine and putrescine, respectively. Concentrations of histamine, tyramine and spermine were below the detection limit (5 ppm). Figure 1A shows the relative abundance of bacterial phyla present in diets collected from the mixing tanks. The most abundant phyla in the mixing tank were *Proteobacteria* (54%), *Cyanobacteria* (38%) and, to a lesser extent, *Firmicutes* (5%), whereas, in the samples collected from the feed troughs in the pig pens *Firmicutes* (77%) dominated followed by *Proteobacteria* (14%) or *Bacteroidetes* (5%) and *Cyanobacteria* (4%). The microbiota composition at phylum level was similar for all dietary treatments when samples from the mixing tanks were investigated. However, a reduction in the relative abundance of *Proteobacteria* was observed in the feed troughs for the soaked diets compared with the fresh diets (19 vs. 8%, Fig. 1A). Figure 1B shows the relative abundance of the 20 most abundant bacterial genera present in the dietary samples analysed. The three most abundant genera in the mixing tanks were *Pseudomonas* (21%), *Pantoea* (15%) and *Acinetobacter* (9%), whereas *Lactobacillus* (42%), *Leuconostoc* (17%), *Weissella* (11%) and *Lactococcus* (9%) predominated in samples collected from the feed troughs. No major differences in the microbial profile were observed at genus level between dietary treatments sampled from either the mixing tanks or feed troughs (Fig. 1B); however, *Lactobacillus* was more abundant in pen trough samples belonging to the soaked dietary treatments than those from the fresh diets (53.6 *vs*. 29.8%, SD 0.20, n = 2).

**Table 2:** Analysed pH, volatile fatty acid concentration (VFA, mmol/kg) and microbial counts (Log_10_ CFU/g) of the dietary treatments collected from the mixing tanks and the feed troughs in the pig pens (n = 2).

*Sampling location:*	Mixing Tank				Pen Troughs				SD^c^
*Cereal soaking (C*_soak_)^a^*:*	Fresh	Fresh	Soak	Soak	Fresh	Fresh	Soak	Soak	
*Enzyme (ENZ)*^b^:	−	+	−	+	−	+	−	+	
**pH**	6.2	6.2	6.2	6.1	5.7	5.0	5.7	5.3	0.03
**VFA**									
Acetate	11.5	13.9	11.8	14.0	22.5	29.7	31.9	28.6	1.15
Propionate	0.2	0.2	0.2	0.2	0.3	0.8	0.4	0.3	0.08
Isobutyrate	1.4	1.5	1.5	1.5	1.1	1.0	1.1	0.9	0.13
Butyrate	0.3	0.2	0.3	0.3	0.3	0.4	0.8	0.3	0.16
Isovalerate	0.1	0.1	0.1	0.1	0.1	0.1	0.1	0.1	0.04
Valerate	0.6	0.6	0.5	0.7	0.3	0.3	0.4	0.3	0.07
Total VFA	14.2	16.4	14.3	16.6	24.6	31.9	35.2	30.7	1.38
Acetate:Propionate ratio	55.1	71.3	60.7	68.5	77.4	80.7	59.5	92.4	10.53
Protein-derived VFA	2.1	2.1	2.1	2.2	1.4	1.4	1.5	1.3	0.17
**Microbial counts**									
Lactic acid bacteria	6.2	6.2	6.4	6.4	8.8	8.7	8.9	9.0	1.52
Enterobacteriaceae	5.8	5.8	5.8	5.7	6.9	6.9	6.4	6.7	1.46
Yeasts	5.2	5.7	5.2	4.6	5.6	6.1	6.6	6.5	1.49
Moulds^d^	<DL	<DL	<DL	<DL	<DL	<DL	<DL	<DL	—

^a^Soaking the cereal fraction of the diet prior to feeding (fresh = 0 h soaking, soak = 3 h soaking).

^b^Enzyme supplementation with a Xylanase and β-glucanase complex [unsupplemented (−), supplemented (+)].

^c^SD = standard deviation.

^d^DL = detection limit (3 Log_10_ CFU/g).

**Table 3:** Analysed nutrient composition and biogenic amine concentrations of dietary treatments collected from the pen troughs (on a 88% DM basis, g/kg unless otherwise stated).

*Cereal soaking (C*_soak_)^a^:	Fresh	Fresh	Soak	Soak
*Enzyme (ENZ)*^b^:	−	+	−	+
Gross energy (MJ/kg)	16.2	16.2	16.2	16.1
Crude fibre	36.1	35.2	35.2	36.1
Neutral detergent fibre	118.8	118.8	121.1	124.1
Acid detergent ibre	44.9	46.6	46.6	47.5
Ash	39.6	40.5	40.3	41.4
Crude protein	162.0	162.0	160.2	164.5
Lysine	8.20	8.97	9.15	9.32
Biogenic amines (ppm)^c^				
Putrescine	<DL	6	<DL	18
Histamine	<DL	<DL	<DL	<DL
Cadaverine	41	89	46	122
Spermidine	26	8	13	10
Tyramine	<DL	<DL	<DL	<DL
Spermine	<DL	<DL	<DL	<DL

^a^Soaking the cereal fraction of the diet prior to feeding (Fresh = 0 h soaking, Soak = 3 h soaking).

^b^Enzyme supplementation with a Xylanase and β-glucanase complex [unsupplemented (−), supplemented (+)].

^c^DL = detection limit (5 ppm).

### Impact of cereal soaking and enzyme dietary supplementation on nutrient digestibility

Results for the apparent ileal digestibility (AiD) and apparent total tract digestibility (ATTD) of DM, organic matter (OM), CP and GE are presented in Table 4. The AiD of DM, OM, CP and GE were unchanged by dietary treatment (P > 0.05). An interaction between C_soak_ and ENZ was observed for ATTD of OM and GE (P < 0.05). Soaking the cereal fraction of the diet improved the ATTD of OM and GE in pigs fed non-ENZ-supplemented diets but soaking did not improve GE or OM ATTD of ENZ-supplemented diets (P < 0.05). The ATTD of DM and CP was improved by dietary supplementation with ENZ (P < 0.001).

**Table 4:** Effect of dietary soaking of cereals with or without carbohydrase supplementation on the coefficient of apparent ileal digestibility (AiD, %), apparent total tract digestibility (ATTD, %), digestible energy (MJ/kg on a DM basis), pH and volatile fatty acid (VFA, mmol/kg) concentrations in the ileal and caecal digesta of grow-finisher pigs^a^.

*Cereal soaking (C*_soak_)^b^:	Fresh	Fresh	Soak	Soak	SEM^d^	*P-*Value		
*Enzyme (ENZ)*^c^:	−	+	−	+		ENZ	C_soak_	ENZ*C_soak_
**AiD**								
Dry matter	62.8	64.2	63.0	63.8	1.70	0.51	0.95	0.87
Organic matter	64.2	66.6	64.3	66.1	1.69	0.22	0.90	0.87
Crude protein	56.5	61.6	59.5	61.1	2.16	0.10	0.59	0.43
Gross energy	60.5	63.0	61.8	62.5	1.81	0.39	0.81	0.62
**ATTD**								
Dry matter	81.7	85.1	83.3	85.1	0.48	<0.001	0.11	0.12
Organic matter	83.0^b^	86.8^a^	84.6^c^	86.7^a^	0.42	<0.001	0.10	<0.05
Crude protein	76.9	84.2	78.8	83.4	0.82	<0.001	0.51	0.13
Gross energy	79.6^c^	83.9^a^	81.8^b^	84.0^a^	0.55	<0.001	<0.05	<0.05
**Digestible energy**	14.8^c^	15.4^a^	15.1^b^	15.4^a^	0.10	<0.001	<0.001	<0.001
**Ileal digesta**								
pH	6.5	6.3	6.3	6.4	0.23	0.74	0.72	0.44
Total VFA^e^	50.7	44.6	47.6	44.3	10.80	0.65	0.86	0.89
**Caecal digesta**								
pH	5.7	5.7	5.6	6.0	0.16	0.13	0.42	0.22
Acetate	92.1	90.0	106.7	86.7	5.44	<0.05	0.29	0.11
Propionate	25.1	21.3	28.5	19.1	2.26	<0.01	0.78	0.20
Isobutyrate	1.4	1.2	1.2	1.1	0.17	0.29	0.47	0.72
Butyrate	19.0	13.8	20.9	13.0	1.68	<0.01	0.78	0.44
Isovalerate	1.0	1.2	1.0	1.2	0.18	0.80	0.27	0.96
Valerate	2.8	2.0	2.7	1.7	0.34	<0.05	0.65	0.75
Total VFA	141.4	129.7	161.5	122.7	9.09	<0.05	0.46	0.14
Acetate:Propionate	3.7	4.3	0.8	6.0	0.83	0.12	0.32	0.37
Protein-derived VFA	5.2	4.1	5.2	4.0	0.45	<0.05	0.86	0.91

^a^Values within a row with a different superscript are statistically different (P < 0.05).

^b^Soaking the cereal fraction of the diet prior to feeding (fresh = 0 h soaking, soak = 3 h soaking).

^c^Enzyme supplementation with a Xylanase and β-glucanase complex [unsupplemented (−), supplemented (+)].

^d^SEM = standard error of the mean.

^e^Concentrations of each individual VFA were not significantly different between treatments, and therefore only the total VFA concentration is shown.

### Impact of cereal soaking and dietary enzyme supplementation on pH and VFA concentrations in the ileal and caecal digesta

The pH and VFA concentrations of ileal and caecal digesta of the pigs fed the different treatments are presented in Table 3. In the ileum, no significant differences were found; neither the total VFA concentration, nor the concentration of each VFA (data not shown), was significantly affected by the interaction ENZ*C_soak_ or the main factors ENZ supplementation or C_soak_. There was no interaction (P > 0.05) between ENZ and C_soak_ for any of the VFAs measured in the caecum. However, supplementation of ENZ to diets reduced caecal concentrations of acetate, propionate, butyrate and valerate. As a consequence, concentrations of total VFAs and protein-derived VFAs in the caecum were significantly lower in pigs fed ENZ-supplemented diets compared to pigs fed non supplemented diets.

### Impact of cereal soaking and enzyme dietary supplementation on pig growth and carcass quality traits

Pig growth and carcass quality traits are presented in Table 5. An interaction between ENZ and C_soak_ was found for average daily gain (ADG) during the growing phase (day 0 to 21; P < 0.05). Pigs fed the Soak + ENZ diet had higher ADG than pigs fed the Fresh + ENZ diet, whereas the ADG of pigs fed Fresh and Soak diets were not statistically different (day 0 to 21). No interaction between C_soak_ and ENZ supplementation was observed for live weight (LW), average daily feed intake (ADFI), feed conversion ratio (FCR) or any of the carcass traits measured at any time period (day 0 to 21, day 21 to 70 or overall; P > 0.05). Pigs fed the soaked diets were 0.8 kg heavier than pigs fed the fresh diets at day 21 (P < 0.05). At slaughter, pigs fed the ENZ-supplemented diets had a greater fat depth (+0.5 mm; P < 0.05) and a lower lean meat percentage (−0.4%; P < 0.05) than pigs fed non-ENZ-supplemented diets.

**Table 5:** Effect of dietary cereal soaking with or without carbohydrase supplementation on growth, feed intake, feed efficiency and carcass quality of grow-finisher pigs^a^.

*Cereal soaking (C*_*soak*_)^b^:	Fresh	Fresh	Soak	Soak	SEM^d^	*P-*value		
*Enzyme (ENZ)*^c^:	−	+	−	+		ENZ	C_soak_	ENZ*C_soak_
**Live weight, kg**								
Day 0	33.5	33.3	33.3	33.5	1.25	0.99	0.96	0.87
Day 21	50.7	50.0	51.1	51.2	0.36	0.36	<0.05	0.23
Day 70	99.0	98.2	98.6	100.4	0.73	0.47	0.23	0.08
**ADFI**^**e**^**, g/day**								
Day 0 to 21	2010	1984	1991	2059	46.7	0.66	0.55	0.67
Day 21 to 70	3033	2975	2976	3041	46.7	0.94	0.92	0.63
Day 0 to 70	2521	2479	2484	2550	43.0	0.78	0.71	0.21
**ADG**^**f**^**, g/day**								
Day 0 to 21	881^a,b^	845^b^	896^a,b^	902^a^	15.3	0.33	<0.05	<0.05
Day 21 to 70	1013	1013	999	1035	15.3	0.23	0.81	0.41
Day 0 to 70	947	929	947	969	11.1	0.87	0.08	0.08
**FCR**^**g**^**, g/g**								
Day 0 to 21	2.36	2.43	2.32	2.38	0.067	0.34	0.48	0.70
Day 21 to 70	3.06	2.97	3.00	3.00	0.067	0.51	0.80	0.84
Day 0 to 70	2.71	2.70	2.66	2.69	0.059	0.86	0.59	0.76
**Carcass traits**								
Carcass cold weight, kg	73.8	73.6	73.5	75.1	0.67	0.31	0.38	0.18
Kill out percentage, %	74.5	74.7	74.7	74.7	0.23	0.59	0.73	0.73
Muscle depth, mm	50.5	49.9	50.5	50.1	0.39	0.18	0.83	0.89
Fat depth, mm	12.6	12.8	12.2	13.0	0.21	<0.05	0.66	0.18
Lean meat, %	57.0	56.9	57.4	56.7	0.18	<0.05	0.56	0.19

^a^Values within a row that do not share a common superscript are statistically different (P < 0.05).

^b^Soaking the cereal fraction of the diet prior to feeding (fresh = 0 h soaking, soak = 3 h soaking).

^c^Enzyme supplementation with a xylanase and β-glucanase complex [unsupplemented (−), supplemented (+)].

^d^SEM = standard error of the mean.

^e^ADFI = average daily feed intake, as 88%DM.

^f^ADG = average daily gain.

^g^FCR = feed conversion ratio, as 88% DM of feed intake.

### Impact of cereal soaking and enzyme dietary supplementation on the intestinal microbiota of pigs

**Figure 2:**
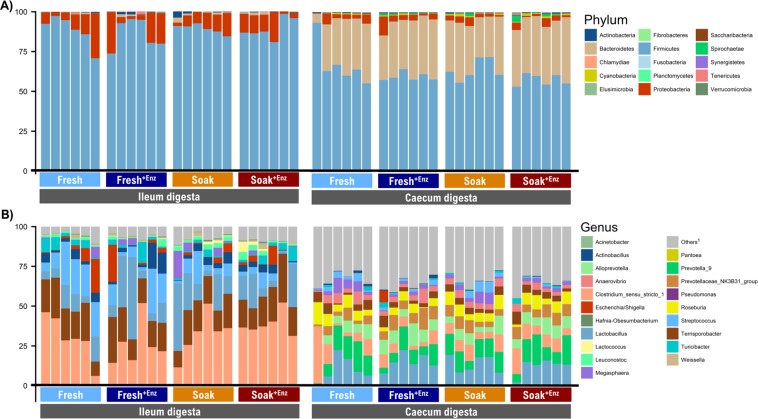
Relative abundance (%) of bacterial phyla (**A**) and the 20 most abundant bacterial genera (**B**) present in the ileal and caecal digesta of pigs fed the experimental dietary treatments. Each bar represents the bacterial profile of the corresponding sample for each individual pig (n = 6/treatment). The dietary treatments were: Fresh = liquid feed prepared with fresh ingredients (); Fresh + ENZ = Liquid fresh feed supplemented with a xylanase and β-glucanase (XB) carbohydrase enzyme (); Soak = Liquid feed prepared with 3h-soaked cereals (); Soak + ENZ = Liquid feed prepared with 3h-soaked cereals supplemented with the XB enzyme (). ^1^Others = All bacterial genera not included in the 20 most abundant.

Alpha and beta diversity of the microbiota of ileal and caecal digesta samples from pigs fed the different treatments were measured, but no statistical differences or clustering according to dietary treatment or main factor were observed (data not shown). Figure 2A shows the relative abundance (%) of bacterial phyla in the ileum and caecum of pigs fed the different dietary treatments. *Firmicutes* (88.1%) and *Proteobacteria* (10.3%) were the two most dominant phyla in the ileal digesta, while *Firmicutes* (61.9%) and *Bacteroidetes* (32.7%) predominated in the caecal digesta. Figure 2B presents the relative abundance (%) of the 20 most abundant bacterial genera observed in the ileum and the caecum of pigs fed the different diets. *Clostridium_sensu_stricto_*1 was the most abundant in the ileal digesta (31.8%), whereas *Lactobacillus* (12.7%) predominated in the caecum.

**Figure 3:**
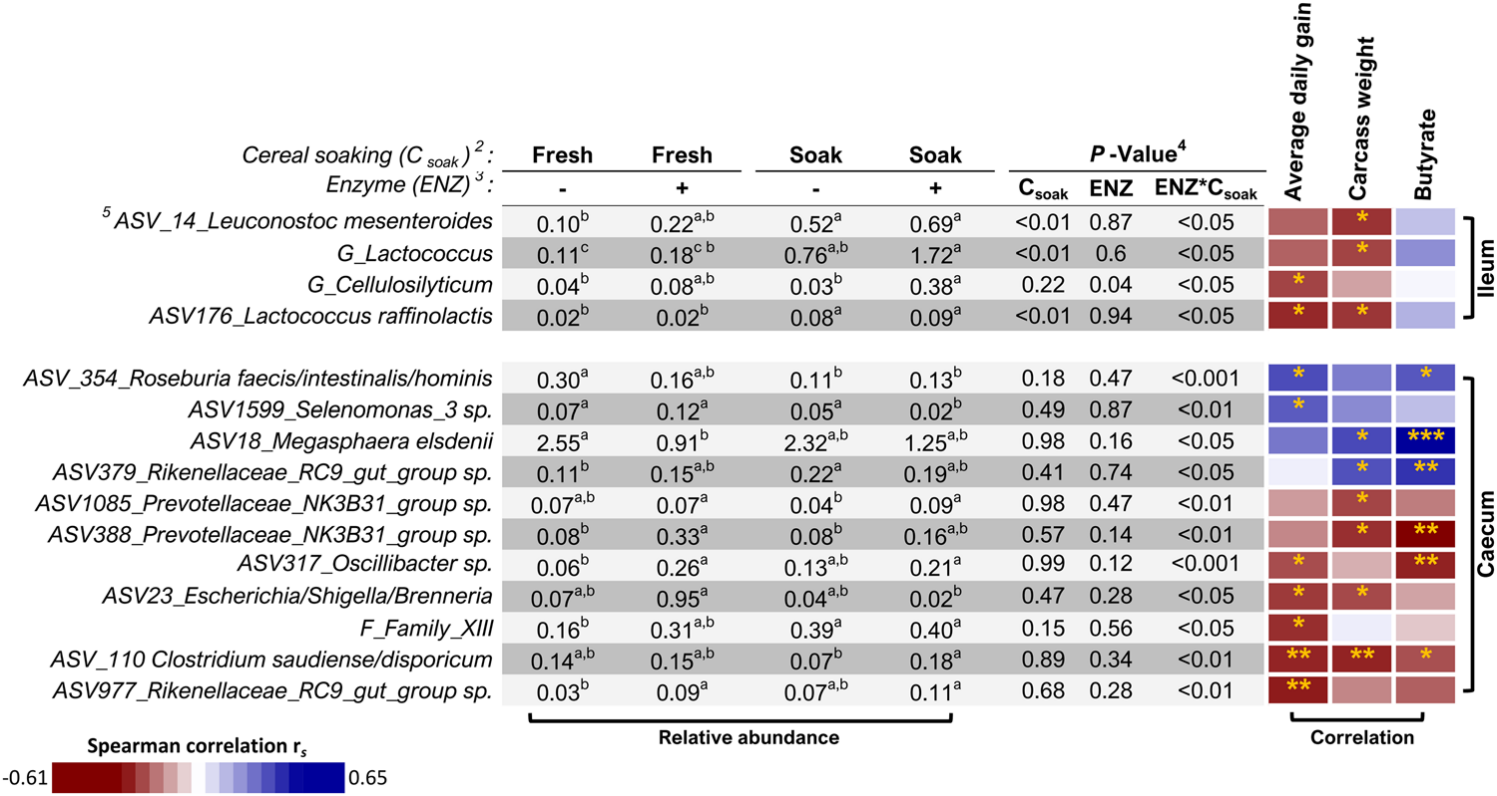
Heatmap showing spearman correlations between the ileal and caecal bacterial taxa found to be differentially abundant between dietary treatments and selected physiological measures in pigs fed the different dietary treatments (n = 6/treatment). The relative abundance (%) of each dietary treatment and the corresponding P-value is shown in the adjacent table. Positive correlations are indicated in blue and negative correlations are indicated in red. Significant correlations are indicated with asterisks (P < 0.05 = *, P < 0.01 = **, P < 0.001 = ***)^1^. ^1^Values within a row that do not share a common superscript are statistically different (P < 0.05). ^2^Soaking the cereal fraction of the diet prior to feeding (fresh = 0 h soaking, soak = 3 h soaking). ^3^Enzyme supplementation with a xylanase and β-glucanase complex [unsupplemented (−), supplemented (+)]. ^4^P-value corrected for false discovery rate (FDR). ^5^*F_* = Family, *G_* = Genus, *ASV_* = Exact amplicon sequence variant.

The relative abundance of phyla, families and genera that were differentially abundant according to dietary treatment (effect of ENZ, C_soak_, or the interaction between ENZ and C_soak_) in the ileal and caecal digesta are presented in Table 6, and the differentially abundant exact ASV are presented in Supplementary Table S1. Differences between dietary treatments were observed in the ileum for 1 phylum, 5 families, 7 genera and 9 ASV, while in the caecum 7 families, 6 genera and 105 ASV were differentially abundant. Spearman correlations between the differentially abundant taxa and all physiological measures recorded in the pigs are presented in Supplementary Figs. S1 and S2 online. In an attempt to understand the treatment-related differences in digestibility but the lack of effect on animal growth, the focus here will be on taxa that were correlated with ADG, carcass weight and/or intestinal butyrate concentration, and these are shown in Fig. 3. It is important to note that the correlations reported indicate an association between bacterial taxa and physiological measures but do not necessarily imply causation. *Megasphaera elsdenni* was reduced in the caecum of pigs fed the Fresh + ENZ diet compared to pigs fed the fresh un-supplemented diet and this ASV was positively correlated with carcass weight and butyrate concentration. The relative abundance of *Prevotellaceae* NK3B31 sp. (ASV388), *Oscillibacter sp*. and *Rikenellaceaceae_RC9* (ASV977) was increased in the caecum of pigs fed the Fresh + ENZ diets compared to pigs fed the fresh un-supplemented diet and these ASV were negatively correlated with growth parameters and butyrate production. The relative abundance of the genus *Cellulolysiticum* in the ileum of the pigs fed the Soak + ENZ diet was increased compared to pigs fed the soaked diet and this genus was negatively correlated with ADG. The relative abundance of an ASV mapped to the *Selenomonas genus* (ASV1599) was decreased in the caecum of pigs fed the Soak + ENZ diet compared to pigs fed the soaked diet and this ASV was positively correlated with ADG. The relative abundance of *Prevotellaceae_NKB31_group sp*. (ASV1085) and *Clostridium saudiense/disporicum* was increased when pigs were fed Soak + ENZ diet compared to pigs fed the soaked diet and these ASV were negatively correlated with carcass weight, ADG, and butyrate, respectively. The relative abundances of *Leuconostoc mesenteroides*, the genus *Lactococcus* and *Lactococcus raffinolactis* were increased in the ileum of pigs fed the soaked diet compared to pigs fed the fresh diet, and all of these LAB were negatively correlated with carcass weight. The relative abundance of *Roseburia faecis/intestinalis/hominis* was decreased in the caecum of pigs fed the soaked diet compared to pigs fed the fresh diet and this ASV was positively correlated with ADG and butyrate concentration. The relative abundance of *Rikenellaceae_RC9* (ASV379) and the family XII was increased in the caecum of pigs fed the soaked diet compared to pigs fed the fresh diet and family XII was negatively correlated with ADG, while *Rikenellaceae_RC9* (ASV379) was positively correlated with butyrate concentration. The relative abundance of the genus *Lactococcus* and *Lactococcus raffinolactis* was increased in the ileum of pigs fed the Soak + ENZ diets compared to pigs fed the Fresh + ENZ diet and these taxa were negatively correlated with carcass weight. The relative abundance of *Selenomonas* (ASV1599) was decreased in the caecum of pigs fed the Soak + ENZ diet compared to pigs fed the Fresh + ENZ diets and this ASV was positively correlated with ADG. The relative abundance of *Escherichia/Shigella/Brenneria* was increased in the caecum of pigs fed the Soak + ENZ diet compared to pigs fed Fresh + ENZ and this ASV was negatively correlated with ADG.

**Table 6:** Relative abundance (%) of microbial taxa differentially abundant according to dietary treatment in the ileal and caecal digesta of pigs^a^.

*Cereal form (*C_soak_)^b^:	Fresh	Fresh	Soak	Soak	SEM^d^	*P*-value^e^		
*Enzyme (ENZ)*^c^:	−	+	−	+		ENZ	C_soak_	ENZ*C_soak_
**Ileum digesta**^**f**^								
*P_Tenericutes*	0.36	0.51	0.04	0.08	0.278	0.67	<0.05	0.10
*F_Ruminococcaceae*	0.02	0.01	0.02	0.01	0.008	<0.05	0.80	0.40
*F_Enterococcaceae*	0.02	0.07	0.04	0.05	0.027	<0.05	0.98	0.39
*F_Neisseriaceae*	0.01	0.04	0.02	0.06	0.026	<0.05	0.55	0.24
*F_Moraxellaceae*	0.04	0.16	0.10	0.44	0.234	<0.05	0.55	0.18
*F_Lachnospiraceae*	0.12^b^	0.15^b^	0.15^b^	1.01^a^	0.332	<0.05	0.06	<0.05
*G_Lelliottia*	0.07	0.04	0.15	0.22	0.063	0.85	<0.05	0.06
*G_Intestinibacter*	0.57^a,b^	0.10^c^	0.18^c,b^	0.89^a^	0.308	0.78	0.72	<0.05
*G_Citrobacter*	0.44	0.41	1.44	1.43	0.360	0.96	<0.001	0.06
*G_Lachnospiraceae_UCG-007*	0.10^b^	0.09^b^	0.05^b^	0.66^a^	0.252	0.07	0.12	<0.05
*G_Cellulosilyticum*	0.04^b^	0.08^a,b^	0.03^b^	0.38^a^	0.130	<0.05	0.22	<0.05
*G_Acinetobacter*	0.02^b^	0.16^a^	0.10^a^	0.43^a^	0.172	0.21	0.32	<0.01
*G_Lactococcus*	0.11^c^	0.18^c,b^	0.76^a,b^	1.72^a^	0.605	0.60	<0.01	<0.01
**Caecum digesta**^**f**^								
*F_Spirochaetaceae*	0.10^b^	0.65^a^	0.45^a,b^	1.14^a^	0.512	0.12	0.42	<0.05
*F_Family_XIII*	0.16^b^	0.31^a,b^	0.39^a^	0.40^a^	0.075	0.56	0.15	<0.05
*F_Bacteroidaceae*	0.02^b^	0.06^a^	0.05^a,b^	0.08^a^	0.015	0.12	0.52	<0.05
*F_Desulfovibrionaceae*	0.02^b^	0.06^a,b^	0.07^a,b^	0.13^a^	0.037	0.14	0.12	<0.05
*F_Rikenellaceae*	1.17^b^	2.12^a,b^	1.87^a,b^	3.03^a^	0.469	0.12	0.21	<0.05
*F_Enterobacteriaceae*	0.17^b^	1.30^a^	0.15^b^	0.14^b^	0.525	0.12	0.09	<0.05
*F_Leuconostocaceae*	0.03^b^	0.08^a,b^	0.19^a^	0.10^a,b^	0.047	0.81	0.11	<0.05
*G_Rikenellaceae_RC9_gut_group*	1.22^b^	2.30^a^	1.94^a,b^	3.17^a^	0.479	0.09	0.40	<0.05
*G_Ruminococcus_2*	0.23^a^	0.10^b^	0.22^a^	0.09^b^	0.042	<0.01	1.00	<0.05
*G_Lachnospiraceae_AC2044_group*	0.23^c,b^	0.96^a^	0.19^c^	0.71^a,b^	0.287	<0.01	0.82	<0.05
*G_Leuconostoc*	0.02^b^	0.05^a,b^	0.13^a^	0.09^a^	0.027	0.76	0.17	<0.05
*G_Lachnospiraceae_UCG-001*	0.87^a^	0.39^c^	0.53^c,b^	0.78^a,b^	0.103	0.51	0.99	<0.05
*G_Lachnoclostridium_12*	0.03^b^	0.02^c^	0.03^a^	0.01^d^	0.000	0.16	0.99	<0.05

^a^Values within a row that do not share a common superscript are statistically different (P < 0.05).

^b^Soaking the cereal fraction of the diet prior to feeding (fresh = 0 h soaking, soak = 3 h soaking).

^c^Enzyme supplementation with a xylanase and β-glucanase complex [unsupplemented (−), supplemented (+)].

^d^SEM = standard error of the mean.

^e^P-value corrected for false discovery rate (FDR).

^f^*P_* = Phylum, *F_* = Family, *G_* = Genus.

## Discussion

One of the nutritional challenges faced with liquid feeding systems is the microbial degradation of valuable nutrients (i.e. vitamins, minerals and AA) from the time that feed is prepared in the mixing tanks until it is ingested by the pig from the trough. The nutrient and microbial composition of feed pre- and post-mixing and from pen troughs was evaluated in this study. Similar feed pH and VFA concentrations were observed across dietary treatments for comparable samples. However, lower pH (5.4 vs. 6.2), higher total VFA concentration (30.6 vs. 15.4 mmol/g), and higher LAB and yeast counts (8.9 vs. 6.3 and 6.2 vs. 5.2 log_10_ CFU/g, respectively) were observed in feed samples collected from the pen troughs compared to those collected from the mixing tanks, demonstrating that spontaneous fermentation had occurred in the troughs between feeds. In fact, several studies have reported similar LAB, yeast and acetate levels in deliberately fermented liquid feed, with LAB counts ranging from 9.2 to 9.6 log_10_ CFU/g^28,36–41^, yeasts present at 5.2 to 7.2 log_10_ CFU/g^28,36–42^ and acetate ranging from 21 to 26 mmol per kg of fermented feed^37,38,43–45^. However, the complete microbial profile of liquid pig feed, to our knowledge, has not been reported to date. Here, we present high-throughput 16S rRNA gene amplicon sequencing data for liquid pig feed. The most obvious differences observed were those between the mixing tanks and the feed troughs. The most abundant phyla in the mixing tanks were *Proteobacteria*, *Cyanobacteria* and *Firmicutes*, with *Pseudomonas* and *Pantoea* being the predominant genera. On the other hand, *Firmicutes* predominated in the feed troughs, followed by *Proteobacteria* and *Cyanobacteria*, and at the genus level there was a shift towards LAB, as *Lactobacillus, Leuconostoc, Weisseilla and Lactococcus* were most abundant, reflecting the data obtained from the culture-based LAB counts. These differences in the microbial profile provide further evidence that spontaneous lactic acid fermentation occurred in the liquid feed residue that remained in feed troughs between feeds. However, as regards, treatment differences, little was found, except that in the feed troughs, *Proteobacteria* was less abundant and *Lactobacillus* more abundant in the soaked diets. This may indicate that carbohydrates favourable for the growth of *Lactobacillus* were released during the 3 h that the cereal fraction of these diets was soaked prior to feeding. It needs to be noted here that chloroplast ASVs could not be removed prior to analysis and because of this are classified within the *Cyanobacteria* phylum in SILVA which might explain the abundance of *Cyanobacteria* in the feed samples.

Because of the spontaneous fermentation which is likely occurring in the feed troughs, bacterial degradation of AA can potentially occur with the resultant formation of biogenic amines, high levels of which are toxic^27,28,36,46^. Cadaverine and putrescine are biogenic amines formed by the decarboxylation of lysine^47,48^. In this study, only the cereal fraction of the diet was soaked in order to minimise the time that synthetic AA (included in the balancer fraction) were in contact with water prior to feeding. This strategy appears effective, as only minimal degradation of lysine was observed (a 12% reduction from the mixing tank to the trough for the fresh diet and reductions were <4% in the rest of the dietary treatments). Despite the well-known toxic effects of high concentrations of biogenic amines in feed and food^49,50^, we failed to find clear guidelines or regulations regarding the maximum acceptable levels in pig feed. However, even with spontaneous fermentation in the troughs, the concentrations of biogenic amines were low compared to the levels normally found in liquid feed on-farm; the maximum concentrations of cadaverine and putrescine observed were 122 and 18 ppm respectively, while Le Treut^51^, in a study comprising liquid feed samples from 33 French farms, reported averages of 192 and 70 ppm, respectively, with maximum levels as high as 1,182 and 310 ppm. Moreover, our results are in line with those from another study where only the cereal fraction of the diet was fermented and the concentrations of cadaverine and putrescine were also low (153 and 75 ppm, respectively)^38^.

Limited improvements in pig growth due to cereal soaking were observed in the current study (i.e. only during the growing phase), indicating that the response is growth stage-dependent. Previous studies reported improvements in pig growth when soaked diets were fed post-weaning^25,52^, however, to our knowledge the effect of feeding cereal soaked diets to grow-finisher pigs has not been reported before. Feeding cereal soaked diets improved the ATTD of OM and GE of grow-finisher pigs in the current study but these improvements were only observed when diets were not supplemented with the enzyme. Despite improvements in the ATTD of DM, OM, GE and CP, improvements in pig growth or feed efficiency were not observed when the enzyme was supplemented to grow-finisher pigs. Likewise, dietary supplementation with xylanase did not improve pig growth when supplemented to soaked diets in other studies^52,53^. These results are in agreement with a recent meta-analysis^21^ where consistent improvements in nutrient digestibility were found over 13 independent XB dietary supplementation experiments, but these differences did not always result in improvements in pig growth or feed efficiency. Diets in the current study were formulated to 7% below the NRC recommended level for NE and the greater GE ATTD together with the increase in carcass backfat thickness observed demonstrates enzyme efficacy.

The intestinal microbiota profile of the pigs reported in this study is, for the most part, in agreement with that reported in the literature, with the three most abundant phyla in the ileal and caecal digesta of our study (*Firmicutes, Proteobacteria and Bacteroidetes*) also found to be the most abundant in a recent meta-analysis defining the core microbiome of pigs^54^. However, some differences were observed at genus level which might be explained, at least in part, by the fact that this is the first study to our knowledge, to report the intestinal microbiota profile of liquid-fed pigs, while the studies included in the meta-analysis were conducted in pigs fed diets in dry form. Nonetheless, there was considerable similarity as regards the predominant genera; in the ileum, *Lactobacillus, Clostridium* and *Terrisporobacter* predominated in our study, while the most abundant genera reported in the aforementioned meta-analysis were *Lactobacillus, Clostridium* and *Streptococcus*. In the caecum, *Prevotella, Streptococcus* and *Clostridium* predominated in our study, while *Prevotella, Escherichia/Shigella* and *Clostridium* were the three most abundant genera in the aforementioned meta-analysis.

In the current study, ileal relative abundance of the LAB, *Leuconostoc mesenteroides* (ASV14), *Lactococcus raffinolactis* (ASV176) and the *Lactococcus* genus was higher in pigs fed the cereal-soaked diets; however, these bacterial taxa were negatively associated with ADG and/or carcass weight. This might help to explain why ileal nutrient digestibility was not increased and also why the results observed for the total tract nutrient digestibility due to cereal soaking of non-enzyme-supplemented pigs were not translated into increased growth performance and improved feed efficiency. Although a number of recent studies have reported bacterial taxa that are associated with body weight (52) and feed efficiency (7, 8, 53, 54) in pigs, to our knowledge, ileal abundance of these bacterial taxa has not been associated with growth parameters to date. However, in line with our findings, a higher abundance of *Lactococcus* was previously observed in the faeces of lighter body weight pigs^55^. In humans, LAB are traditionally seen as having a positive impact on the host^56^. However, our results highlight that this does not necessarily mean that these bacterial groups improve growth in production animals. The other bacterial taxon found to be differentially abundant in the ileum was *Cellulosilyticum*, which, although at low relative abundance, was enriched in enzyme-supplemented pigs fed C_soak_ diets and also negatively associated with pig growth. However, in other studies the relative abundance of *Cellulosilyticum lentocellum* in the jejunum was positively associated with lean pigs^57^, and the faecal relative abundance of the *Cellulosilyticum* genus was higher in pigs with better feed efficiency^3^.

*Selenomonas sp*. (ASV1599) and *Megasphaera elsdenii* (ASV18) were lower in relative abundance in the caecum of pigs fed the enzyme-supplemented diets (*M. elsdenii* was reduced when the fresh diet was supplemented and *Selenomonas sp*. (ASV1599) when the soaked diet was supplemented). It has been reported that some *Megasphaera* species are unable to metabolize xylose (58) (the product of xylanase activity); therefore, it is possible that supplementation of the XB enzyme complex in the current study favoured the growth of microbial taxa more adapted to xylose utilisation. As regards the effects of cereal soaking, *Roseburia faecis/intestinalis/hominis* (ASV354) decreased in abundance when the Fresh diet was soaked, while *Rikenellaceae*_RC9_gut_group sp. (ASV379) increased. These four differentially abundant taxa in the caecum of pigs (*Megasphaera elsdenii, Selenomonas sp*., Roseb*uria faecis/intestinalis/hominis* and *Rikenellaceae*_RC9_gut_group sp.) were the only taxa positively associated with pig growth in this study. As shown, these four taxa had to some extent a lower relative abundance in pigs fed the treatment diets (C_soak_ or ENZ supplemented). With the exception of *Selenomonas sp*. (ASV1599), all of them were also positively associated with caecal butyrate concentration. This is in agreement with a previous study where *Megasphaera elsdenii*, *Roseburia faecis*, and *Roseburia hominis* were identified as butyrate-producing within the GIT of pigs^58^. Butyrate production in the caecum is associated with improved gut health, reduced proliferation of pathogenic bacteria and also contributes to the animal’s energy balance as it is metabolised by colonic epithelial cells^59^. In line with this, some of the taxa that were negatively associated with growth parameters were also negatively associated with butyrate concentration in the caecum (*Prevotellaceae_NK3B31*, *Oscillibacter sp*., and *Clostridium saudiense/disporicum*) and these taxa were higher in abundance in the caecum of pigs fed the enzyme-supplemented diets. These results may help to explain why improvements in growth and feed efficiency were not found in pigs fed enzyme-supplemented liquid diets despite the observed improvements in nutrient digestibility. In agreement with our results, *Prevotellaceae*, *Clostridium, Oscillibacter* and *Rikenellaceae* operational taxonomic groups were more abundant in the caecum of pigs with low feed efficiency in previous studies^60,61^. It is also interesting to note, that the taxa with higher abundance in the caecum of enzyme-supplemented pigs differed between those fed fresh and soaked diets; *Prevotellaceae*_NK3B31 sp. (ASV388), *Oscillibacter* sp. (ASV317) and *Rikenellaceae*_RC9 sp. (ASV977) were increased when the fresh diets were supplemented with enzyme, whereas, *Prevotellaceae* NK3B31 sp. (ASV1085) and *Clostridium saudiense/disporicum* (ASV110) were increased when the soaked diets were supplemented with enzyme. A possible explanation for this might be that the 3 h soaking time allowed the enzyme complex to begin degradation of substrates prior to feed delivery and this was reflected in the caecum of pigs fed the soaked diets.

## Conclusions

In conclusion, despite evidence of spontaneous fermentation of liquid feed in the pen troughs, minimal degradation of dietary AA resulted. Soaking the cereal fraction of the diet increased pig growth during the early grow-finisher period but not thereafter. Enzyme supplementation increased total tract nutrient digestibility and reduced caecal VFA concentrations but did not improve pig growth or feed efficiency. Cereal soaking and enzyme supplementation modulated the intestinal microbiota composition of liquid-fed pigs; however, our data shows that both strategies promoted the abundance of bacterial taxa that were negatively associated with pig growth and reduced the abundance of taxa positively associated with pig growth and butyrate concentration in the caecum. This may help to explain the lack of consistency observed between nutrient digestibility and pig growth results when feed enzymes are supplemented to liquid-fed pigs. Additional research is needed to investigate the reliability of the associations between growth and microbial taxa identified here. The findings of this study can be used to design intervention studies where, depending on culturability, the growth-associated taxa are isolated and tested *in-vivo*.

### Consent for publication

All authors critically revised the manuscript for important intellectual content and approved the final manuscript.

## Supplementary information

Supplementary material.

## Data Availability

The datasets used and/or analysed during the current study are available from the corresponding author on reasonable request.

## References

[1] Clark M, Tilman D (2017). Comparative analysis of environmental impacts of agricultural production systems, agricultural input efficiency, and food choice. Env. Res. Lett..

[2] Aarnink AJA, Verstegen MWA (2007). Nutrition, key factor to reduce environmental load from pig production. Livest. Sci..

[3] McCormack UM (2017). Exploring a possible link between the intestinal microbiota and feed efficiency in pigs. Appl. Environ. Microbiol..

[4] Vigors S, O’Doherty JV, Kelly AK, O’Shea CJ, Sweeney T (2016). The Effect of Divergence in Feed Efficiency on the Intestinal Microbiota and the Intestinal Immune Response in Both Unchallenged and Lipopolysaccharide Challenged Ileal and Colonic Explants. PloS one..

[5] Verschuren LMG (2018). Fecal microbial composition associated with variation in feed efficiency in pigs depends on diet and sex. J. Anim. Sci..

[6] Flint HJ, Scott KP, Duncan SH, Louis P, Forano E (2012). Microbial degradation of complex carbohydrates in the gut. Gut Microbes.

[7] Jensen BB, Jørgensen H (1994). Effect of dietary fiber on microbial activity and microbial gas production in various regions of the gastrointestinal tract of pigs. Appl. Environ. Microbiol..

[8] Konstantinov SR (2003). Effect of fermentable carbohydrates on piglet faecal bacterial communities as revealed by denaturing gradient gel electrophoresis analysis of 16S ribosomal DNA. FEMS Microbiol. Ecol..

[9] Willamil J, Badiola I, Devillard E, Geraert P, Torrallardona D (2012). Wheat-barley-rye-or corn-fed growing pigs respond differently to dietary supplementation with a carbohydrase complex. J. Anim. Sci..

[10] Agyekum A (2015). Effect of supplementing a fibrous diet with a xylanase and β-glucanase blend on growth performance, intestinal glucose uptake, and transport-associated gene expression in growing pigs. J. Anim. Sci..

[11] Barrera M, Cervantes M, Sauer W, Araiza A, Torrentera N (2004). Ileal amino acid digestibility and performance of growing pigs fed wheat-based diets supplemented with xylanase. J. Anim. Sci..

[12] Clarke LC (2018). Effect of β-glucanase and β-xylanase enzyme supplemented barley diets on nutrient digestibility, growth performance and expression of intestinal nutrient transporter genes in finisher pigs. Anim. Feed. Sci. Technol..

[13] Lan R, Li T, Kim I (2017). Effects of xylanase supplementation on growth performance, nutrient digestibility, blood parameters, fecal microbiota, fecal score and fecal noxious gas emission of weaning pigs fed corn-soybean meal-based diet. Anim. Sci. J..

[14] O’Connell JM, Sweeney T, Callan JJ, O’Doherty JV (2005). The effect of cereal type and exogenous enzyme supplementation in pig diets on nutrient digestibility, intestinal microflora, volatile fatty acid concentration and manure ammonia emissions from finisher pigs. Anim. Sci..

[15] Reilly P (2010). The effect of cereal-derived beta-glucans and exogenous enzyme supplementation on intestinal microflora, nutrient digestibility, mineral metabolism and volatile fatty acid concentrations in finisher pigs. Anim. Feed Sci. Technol..

[16] Garry B, Fogarty M, Curran. T, O’Connell M, O’Doherty JV (2007). The effect of cereal type and enzyme addition on pig performance, intestinal microflora, and ammonia and odour emissions. Anim..

[17] Thacker P, Rossnagel B (2005). Effect of enzyme supplementation on the performance of growing-finishing pigs fed diets containing normal or high fat oat. J. Anim. Vet. Adv..

[18] Thacker PA, Campbell GL, Grootwassink J (1992). The effect of organic acids and enzyme supplementation on the performance of pigs fed barley-based diets. Can. J. Anim. Sci..

[19] Thacker PA (2001). Effect of enzyme supplementation on the performance of growing-finishing pigs fed barley-based diets supplemented with soybean meal or canola meal. Asian-Australas. J. Anim. Sci..

[20] Kiarie E, Owusu-Asiedu A, Peron A, Simmins P, Nyachoti C (2012). Efficacy of xylanase and β-glucanase blend in mixed grains and grain co-products-based diets for fattening pigs. Livest. Sci..

[21] Torres-Pitarch A, Manzanilla EG, Gardiner GE, Doherty JVO, Lawlor PG (2019). Systematic review and meta-analysis on the effect of feed enzymes on growth and nutrient digestibility in grow-finisher pigs: effect of enzyme type and cereal source. Anim. Feed. Sci. Technol..

[22] Basinskiene, L., Juodeikiene, G., Kalvaityte, V., Ceseviciene, J. & Leistrumaite A. Enzyme activity of different cereals grown using organic and conventional agricultural practices, in *Conference Proceedings of the 6th Baltic Conference on Food Science and* Technology (ed. Straumite E.), 27–32 (2011).

[23] Brijs, K. *et al*. Enzymes and Enzyme Inhibitors Endogenous to Wheat, in *Grain Science References, Wheat*: *Chemistry and Technology* (ed. Kan, K. & Shewry, P. R.), 401–435 (AACC International, 2009).

[24] Larsen T, Skoglund E, Sandberg A-S, Engberg RM (1999). Soaking and pelleting of pig diets alters the apparent absorption and retention of minerals. Can. J. Anim. Sci..

[25] Choct M, Selby EAD, Cadogan DJ, Campbell RG (2004). Effect of liquid to feed ratio, steeping time, and enzyme supplementation on the performance of weaner pigs. Aust. J. Agric. Res..

[26] Liu J, Bollinger DW, Ledoux DR, Ellersieck MR, Veum TL (1997). Soaking increases the efficacy of supplemental microbial phytase in a low-phosphorus corn-soybean meal diet for growing pigs. J. Anim. Sci..

[27] Niven SJ, Beal JD, Brooks PH (2006). The effect of controlled fermentation on the fate of synthetic lysine in liquid diets for pigs. Anim. Feed Sci. Technol..

[28] Canibe N, Jensen BB (2010). Fermented liquid feed - Feed processing has a big impact on microbial degradation of free lysine during fermentation. Livest. Sci..

[29] Lee, L. S. *et al*. National Research Council, Nutrient Requirements of Swine: Eleventh Revised Edition. National Research Concil (NRC), The National Academies Press, Washington, DC, 10.17226/13298 (2012).

[30] Torres-Pitarch A (2018). Effect of phytase, carbohydrase, and protease addition to a wheat distillers dried grains with solubles and rapeseed based diet on *in vitro* ileal digestibility, growth, and bone mineral density of grower-finisher pigs. Livest. Sci..

[31] Iwaki Kazuo, Nimura Noriyuki, Hiraga Yayoi, Kinoshita Toshio, Takeda Kazuyoshi, Ogura Haruo (1987). Amino acid analysis by reversed-phase high-performance liquid chromatography. Journal of Chromatography A.

[32] McCarthy JF, Bowland JP, Aherne FX (1977). Influence of method upon the determination of apparent digestibility in the pig. Can. J. Anim. Sci..

[33] Buzoianu SG (2012). High-Throughput Sequence-Based Analysis of the Intestinal Microbiota of Weanling Pigs Fed Genetically Modified MON810 Maize Expressing Bacillus thuringiensis Cry1Ab (Bt Maize) for 31 Days. Appl. Environ. Microbiol..

[34] Fouhy F (2015). The Effects of Freezing on Faecal Microbiota as Determined Using MiSeq Sequencing and Culture-Based Investigations. PloS one.

[35] R Core Team. R: A language and Environment for Statistical Computing. R Fundation for Statistical Computing, Vienna, Austria, https://www.R-project.org/ (2015).

[36] Canibe N, Virtanen E, Jensen BB (2007). Microbial and nutritional characteristics of pig liquid feed during fermentation. Anim. Feed Sci. Technol..

[37] Canibe N, Jensen BB (2003). Fermented and nonfermented liquid feed to growing pigs: Effect on aspects of gastrointestinal ecology and growth performance. J. Anim. Sci..

[38] Canibe N, Hojberg O, Badsberg JH, Jensen BB (2007). Effect of feeding fermented liquid feed and fermented grain on gastrointestinal ecology and growth performance in piglets. J. Anim. Sci..

[39] Canibe N, Miettinen H, Jensen BB (2008). Effect of adding Lactobacillus plantarum or a formic acid containing-product to fermented liquid feed on gastrointestinal ecology and growth performance of piglets. Livest. Sci..

[40] Moran CA, Scholten RHJ, Tricarico JM, Brooks PH, Verstegen MWA (2006). Fermentation of wheat: Effects of backslopping different proportions of pre-fermented wheat on the microbial and chemical composition. Arch. Anim. Nutr..

[41] Geary TM, Brooks PH, Beal JD, Campbell A (1999). Effect on weaner pig performance and diet microbiology of feeding a liquid diet acidified to pH 4 with either lactic acid or through fermentation with Pediococcus acidilactici. J. Sci. Food. Agric..

[42] Gori K, Bjorklund MK, Canibe N, Pedersen AO, Jespersen L (2011). Occurrence and Identification of Yeast Species in Fermented Liquid Feed for Piglets. Microb. Ecol..

[43] Canibe N, Virtanen E, Jensen BB (2007). Effect of acid addition to pig liquid feed on its microbial and nutritional characteristics. Livest. Sci..

[44] Jakobsen GV, Jensen BB, Knudsen KEB, Canibe N (2015). Fermentation and addition of enzymes to a diet based on high-moisture corn, rapeseed cake, and peas improve digestibility of nonstarch polysaccharides, crude protein, and phosphorus in pigs. J. Anim. Sci..

[45] Beal JD, Niven SJ, Brooks PH, Gill BP (2005). Variation in short chain fatty acid and ethanol concentration resulting from the natural fermentation of wheat and barley for inclusion in liquid diets for pigs. J. Sci. Food Agric..

[46] Brooks P, Beal J, Niven S (2001). Liquid feeding of pigs: potential for reducing environmental impact and for improving productivity and food safety. Recent Adv. Anim. Nutr. Aust..

[47] Ma W (2017). Advances in cadaverine bacterial production and its applications. Engineering.

[48] Romano A, Trip H, Lolkema JS, Lucas PM (2013). Three-component lysine/ornithine decarboxylation system in Lactobacillus saerimneri 30a. J. Bacteriol..

[49] Shalaby AR (1996). Significance of biogenic amines to food safety and human health. Food Res. Int..

[50] Spano G (2010). Biogenic amines in fermented foods. Eur. J. Clin. Nutr..

[51] Treut YL (2012). Biogenic amines in pig liquid feed: Myth or reality?. Pig Progress.

[52] L’Anson KA, Choct M, Brooks PH (2013). Effect of xylanase supplementation of wheat-based liquid diets, for weaner pigs, steeped for 1 or 24 h before feeding. Anim. Prod. Sci..

[53] L’Anson KA, Choct M, Brooks PH (2013). Effect of feed processing and enzyme supplementation on diet digestibility and performance of male weaner pigs fed wheat-based diets in dry or liquid form. Anim. Prod. Sci..

[54] Holman DB, Brunelle BW, Trachsel J, Allen HK (2017). Meta-analysis to define a core microbiota in the swine gut. mSystems.

[55] Han GG (2017). Evaluating the association between body weight and the intestinal microbiota of weaned piglets via 16S rRNA sequencing. Appl. Microbiol. Biotechnol..

[56] Masood MI, Qadir MI, Shirazi JH, Khan IU (2011). Beneficial effects of lactic acid bacteria on human beings. Crit. Rev. Microbiol..

[57] Yang H (2016). Uncovering the composition of microbial community structure and metagenomics among three gut locations in pigs with distinct fatness. Sci. Rep..

[58] Levine UY, Looft T, Allen HK, Stanton TB (2013). Butyrate-Producing Bacteria, Including Mucin Degraders, from the Swine Intestinal Tract. Appl. Environ. Microbiol..

[59] Bedford A, Gong J (2017). Implications of butyrate and its derivatives for gut health and animal production. Anim. Nutr..

[60] Quan J (2018). A global comparison of the microbiome compositions of three gut locations in commercial pigs with extreme feed conversion ratios. Sci. Rep..

[61] Tan Z (2017). Metagenomic Analysis of Cecal Microbiome Identified Microbiota and Functional Capacities Associated with Feed Efficiency in Landrace Finishing Pigs. Front. Microbiol..

