# Supplementary material for: Effect of cereal soaking and carbohydrase supplementation on growth, nutrient digestibility and intestinal microbiota in liquid-fed grow-finishing pigs
Source: Sci Rep. 2020 Jan 23;10:1023. doi: 10.1038/s41598-020-57668-6 (PMC6978375; doi:10.1038/s41598-020-57668-6)
Supplement: Supplementary file 1 — Supplementary material. [file 41598_2020_57668_MOESM1_ESM.pdf]

Supporting information for:

"Effect of cereal soaking and carbohydrase supplementation on growth, nutrient digestibility and intestinal microbiota in liquid-fed grow-finishing pigs"

Alberto Torres-Pitarch[1,2], Gillian E. Gardiner[3], Paul Cormican[4], Mary Rea[5,6], Fiona Crispie[5,6], John V. O'Doherty[2], Pierre Cozannet[7], Tomas Ryan[1], and Peadar G. Lawlor[1*]

[1]Teagasc, Pig Development Department, Animal and Grassland Research and Innovation Centre, Moorepark, Fermoy, County Cork, Ireland

[2]School of Agriculture and Food Science, University College Dublin, Belfield, Dublin, Ireland

[3]Department of Science, Waterford Institute of Technology, Waterford, Ireland

[4]Animal and Bioscience Research Department, Animal and Grassland Research and Innovation Centre, Teagasc, Grange, County Meath, Ireland

[5]Teagasc Food Research Centre, Moorepark, Fermoy, County Cork, Ireland

[6]APC Microbiome Ireland, University College Cork, Cork, Ireland

[7]Adisseo France SAS, Antony, France

[*]Correspondence: peadar.lawlor@teagasc.ie

Supplementary Figure S1

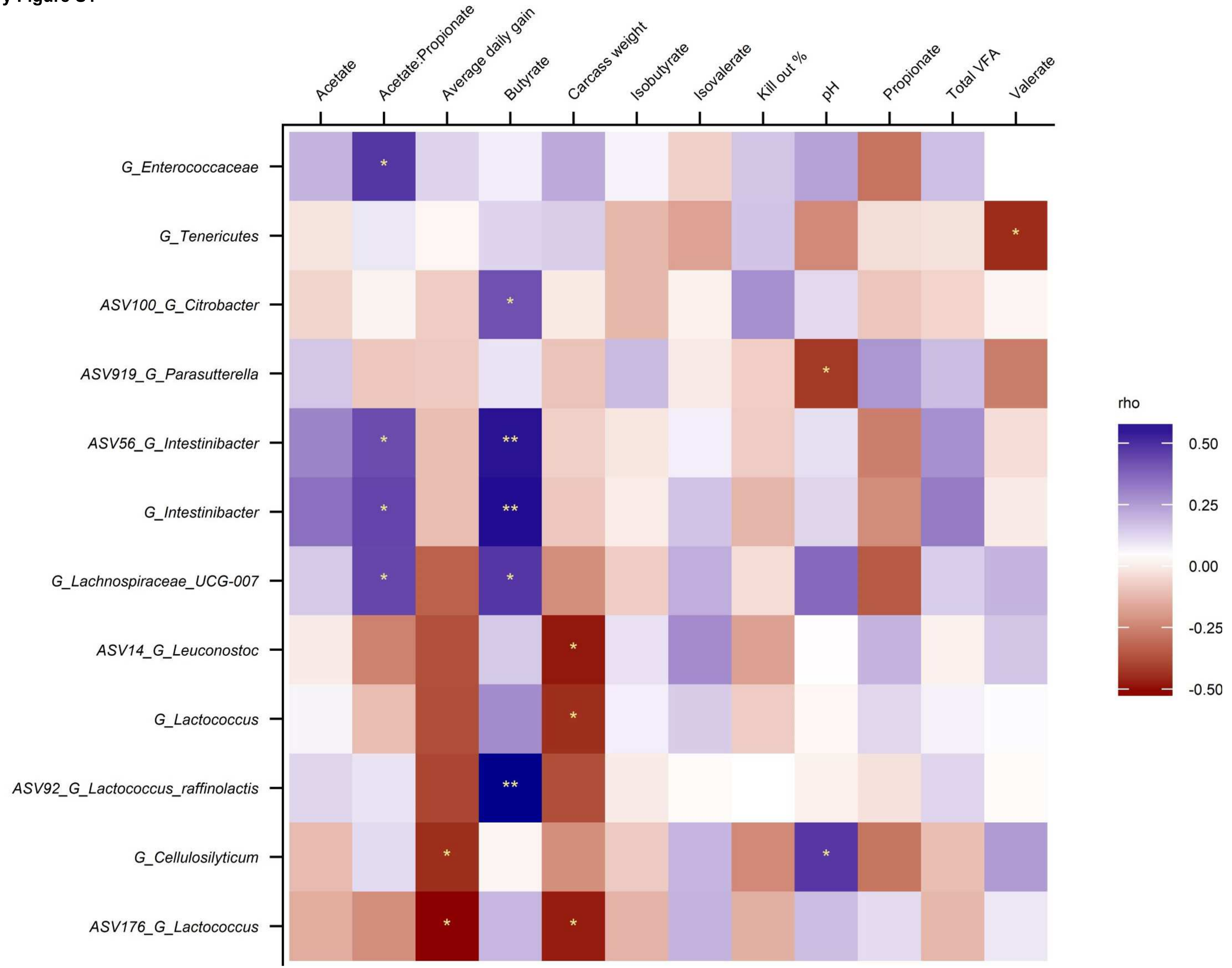

Supplementary Figure S1. Spearman correlation between all ileal taxa with differential relative abundance and all variables measured in the pigs. Positive correlations are indicated in blue and negative correlations are indicated in red. Significant correlations are indicated with asterisks (P<0.05 = *, P<0.01 = **, P<0.001 = ***).

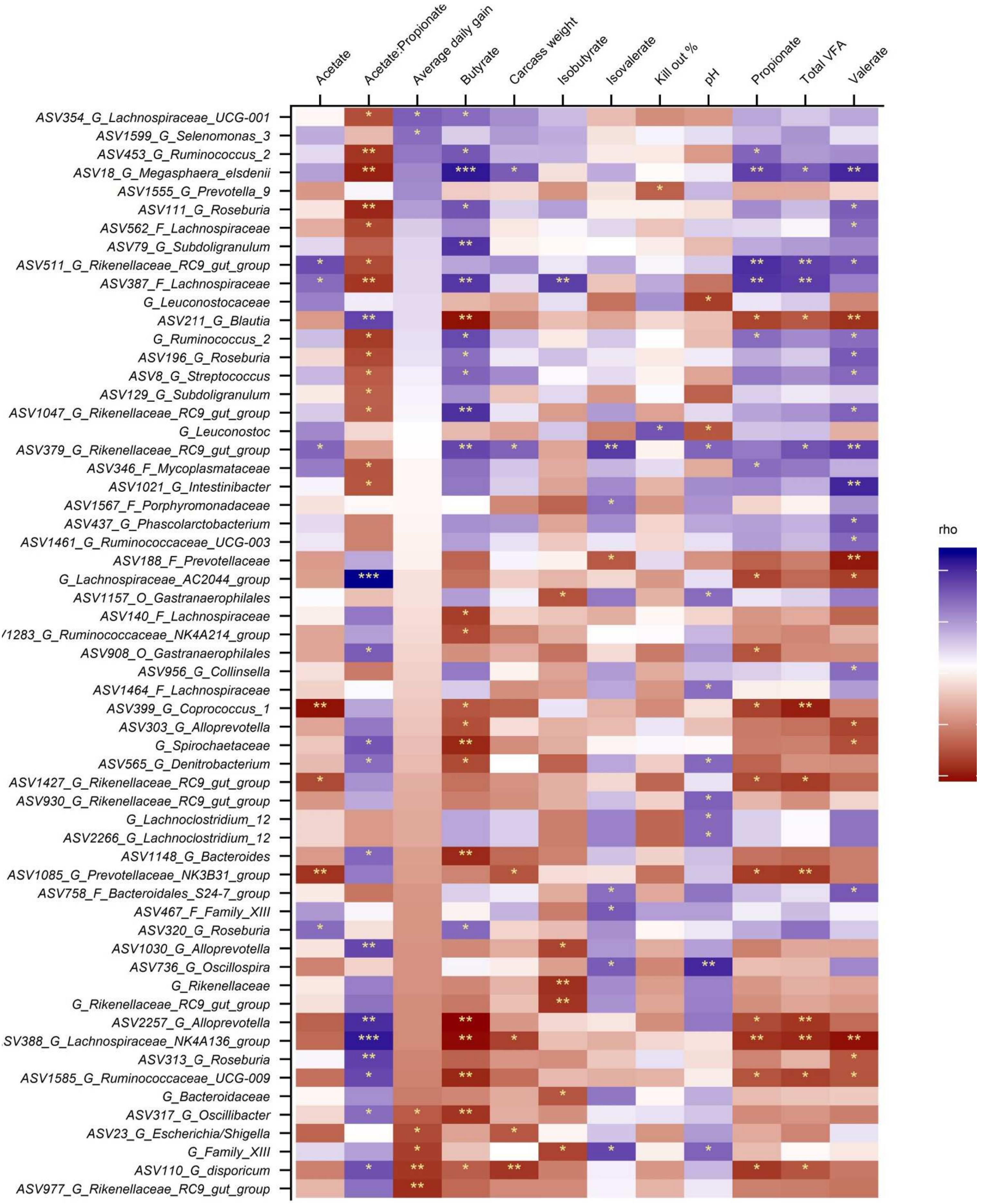

Supplementary Figure S2. Spearman correlation between all caecal taxa with differential relative abundance and all variables measured in the pigs. Positive correlations are indicated in blue and negative correlations are indicated in red. Significant correlations are indicated with asterisks (P<0.05 = *, P<0.01 = **, P<0.001 = ***).

Table S1 Relative abundance (%) of microbial amplicon sequence variants (ASV) differentially abundant according to dietary treatment in the ileal and caecal digesta of pigs (.xls file)[1]

Cereal form (C_{soak}) [2] *:*	Fresh	Fresh	Soak	Soak		***P*-value**[5]		
Enzyme (ENZ) [3] *:*	-	+	-	+	SEM[4]	ENZ	C_{soak}	ENZ*C_{soak}
**Ileum**								
ASV176	0.02b	0.02b	0.08a	0.09a	0.020	<0.001	0.940	0.030
ASV92	0.05b	0.04b	0.17a	0.21a	0.044	<0.001	0.890	<0.001
ASV100	0.12b	0.09b	0.27a	0.25a	0.040	<0.001	0.880	<0.001
ASV56	0.56a,b	0.06c	0.15c b	0.83a	0.282	0.700	0.880	<0.001
ASV66	0.06b	0.11b	0.46a	0.55a	0.163	<0.001	0.880	<0.001
ASV14	0.1b	0.22a,b	0.52a	0.69a	0.206	<0.001	0.870	0.030
ASV919	0.01b	0 c	0.03b	0.1a	0.028	0.070	0.870	<0.001
ASV186	0.09a,b	0.05b	0.15a	0.12a	0.023	0.070	0.580	0.030
ASV22	0.04c	0.11c b	0.32a,b	1.11a	0.496	<0.001	0.400	<0.001
**Caecum**								
ASV1567	0.03a,b	0.04a,b	0.06a	0.02b	0.017	0.920	0.740	0.040
ASV984	0.01a	0.23a	0.01a	0.04a	0.080	0.530	0.240	0.040
ASV1157	0.06a,b	0.02b	0.1a	0.08a,b	0.024	0.500	0.560	0.040
ASV1659	0.02a	0.03a	0.03a	0.05a	0.007	0.410	0.390	0.040
ASV1174	0.03b	0.04a,b	0.09a	0.05a,b	0.016	0.410	0.590	0.040
ASV884	0.08a,b	0.06a,b	0.06b	0.16a	0.034	0.790	0.530	0.040
ASV110	0.14a,b	0.15a,b	0.07b	0.18a	0.038	0.890	0.340	0.040
ASV1203	0.02a	0.47a	0.1a	0.09a	0.104	0.660	0.550	0.040
ASV1070	0.06a	0.05a	0.12a	0.04a	0.013	0.570	0.510	0.040
ASV1153	0.04a,b	0.03b	0.06a	0.05a,b	0.009	0.410	0.490	0.040
ASV1151	0.11a	0.04a,b	0.07a,b	0.01b	0.038	0.840	0.280	0.040
ASV237	0.11b	0.69a	0.17a,b	0.2a,b	0.260	0.590	0.280	0.040
ASV1074	0.05b	0.12a	0.07a,b	0.06a,b	0.022	0.680	0.390	0.040
ASV1890	0.01a	0.04a	0.02a	0.03a	0.011	0.980	0.260	0.040
ASV1014	0.05a	0.03a	0.08a	0.09a	0.022	0.410	0.960	0.030
ASV760	0.03a	0.03a	0.06a	0.09a	0.027	0.320	0.640	0.030
ASV326	0.09a	0.21a	0.17a	0.21a	0.042	0.790	0.320	0.030
ASV1863	0.05a	0.02a	0.04a	0.02a	0.004	0.990	0.120	0.030
ASV256	0.28a	0.12a	0.18a	0.26a	0.064	0.860	0.710	0.030
ASV188	0.16b	0.37a	0.31a,b	0.24a,b	0.069	0.970	0.630	0.030
ASV1427	0.03a,b	0.04a,b	0.02b	0.05a	0.007	0.990	0.240	0.030
ASV1096	0.03b	0.03a,b	0.07a	0.05a,b	0.012	0.330	0.590	0.030
ASV1660	0.03a,b	0.03a,b	0.14a	0.02b	0.032	0.530	0.530	0.030
ASV440	0.14a	0.29a	0.09a	0.12a	0.055	0.410	0.440	0.030
ASV1021	0.08a	0.05a,b	0.07a,b	0.04b	0.007	0.680	0.280	0.030
ASV511	0.08b	0.13a,b	0.14a	0.12a,b	0.021	0.500	0.640	0.030
ASV1283	0.02b	0.08a	0.06a,b	0.07a,b	0.024	0.980	0.340	0.030
ASV1460	0.04a	0.09a	0.03a	0.03a	0.011	0.410	0.620	0.030
ASV970	0.06a,b	0.06a,b	0.16a	0.05b	0.023	0.550	0.350	0.030
ASV565	0.07b	0.11a,b	0.1a,b	0.16a	0.022	0.410	0.290	0.030

Cereal form (C_{soak}) [2] *:*	**Fresh**	**Fresh**	**Soak**	**Soak**		***P*-value** [5]		
Enzyme (ENZ) [3] *:*	**-**	**+**	**-**	**+**	**SEM** [4]	**ENZ**	C_{soak}	**ENZ*** C_{soak}
ASV23	0.07a,b	0.95a	0.04a,b	0.02b	0.452	0.470	0.280	0.030
ASV359	0.15a	0.05a	0.09a	0.03a	0.029	0.810	0.240	0.030
ASV18	2.55a	0.91b	2.32a,b	1.25a,b	0.619	0.980	0.160	0.030
ASV471	0.2a	0.1a	0.16a	0.09a	0.027	0.800	0.140	0.030
ASV933	0.06a,b	0.1a,b	0.05b	0.12a	0.025	0.680	0.140	0.030
ASV658	0.09a,b	0.08a,b	0.07b	0.18a	0.039	0.850	0.380	0.030
ASV303	0.11b	0.21a,b	0.11a,b	0.7a	0.253	0.410	0.240	0.030
ASV1555	0.01b	0.02a,b	0.08a	0.03a,b	0.029	0.410	0.920	0.020
ASV812	0.12a	0.07a,b	0.05b	0.06a,b	0.013	0.410	0.550	0.020
ASV424	0.08b	0.1a,b	0.14a,b	0.2a	0.035	0.330	0.600	0.020
ASV202	0.19a,b	0.47a	0.24a,b	0.16b	0.100	0.540	0.470	0.020
ASV1047	0.04a,b	0.01b	0.07a	0.03a,b	0.018	0.500	0.280	0.020
ASV607	0.22a	0.06a	0.06a	0.03a	0.050	0.410	0.280	0.020
ASV8	1.39a,b	0.77a,b	2.02a	0.36b	0.738	0.980	0.240	0.020
ASV28	1.26a	0.44a	1.22a	0.52a	0.336	0.990	0.120	0.020
ASV863	0a	0.24a	0.26a	0.2a	0.137	0.980	0.780	0.020
ASV379	0.11b	0.15a,b	0.22a	0.19a,b	0.038	0.410	0.740	0.020
ASV703	0.6a	0.08b	0.15a,b	0.14a,b	0.255	0.970	0.280	0.020
ASV907	0.03b	0.09a,b	0.05a,b	0.09a	0.026	0.840	0.270	0.020
ASV387	0.13a,b	0.09a,b	0.18a	0.08b	0.025	0.760	0.160	0.020
ASV166	0.18a	0.1a	0.16a	0.09a	0.029	0.950	0.120	0.020
ASV134	0.53a,b	0.27b	0.64a,b	0.85a	0.181	0.450	0.950	0.010
ASV758	0.06a,b	0.1a	0.08a	0.02b	0.017	0.910	0.760	0.010
ASV1461	0.06a	0.02b	0.03a,b	0.02b	0.008	0.680	0.280	0.010
ASV611	0.05b	0.18a	0.08a,b	0.14a,b	0.046	0.970	0.160	0.010
ASV1464	0.05a	0.01b	0.04a,b	0.06a	0.018	0.730	0.800	0.010
ASV399	0.17a,b	0.24a	0.12a,b	0.1b	0.035	0.320	0.640	0.010
ASV1492	0.01a	0.05a	0.05a	0.04a	0.016	0.620	0.530	0.010
ASV977	0.03b	0.09a	0.07a,b	0.11a	0.017	0.680	0.280	0.010
ASV453	0.14a	0.05b	0.12a,b	0.07a,b	0.029	0.990	0.120	0.010
ASV771	0.02b	0.1a	0.11a	0.12a	0.038	0.660	0.640	0.010
ASV196	0.56a	0.13b	0.26a,b	0.24a,b	0.126	0.620	0.280	0.010
ASV467	0.07b	0.13a,b	0.14a,b	0.2a	0.029	0.410	0.470	0.010
ASV736	0.08a,b	0.12a	0.05b	0.07a,b	0.018	0.410	0.450	0.010
ASV388	0.08b	0.33a	0.08b	0.16a,b	0.079	0.570	0.140	0.010
ASV859	0.07a,b	0.12a	0.14a	0.06b	0.016	0.990	0.680	0.010
ASV1030	0.03b	0.15a	0.07a,b	0.06a,b	0.034	0.610	0.470	0.010
ASV1085	0.07a,b	0.07a	0.04b	0.09a	0.006	0.980	0.470	0.010
ASV270	0.11b	0.16a,b	0.17a,b	0.41a	0.105	0.410	0.280	0.010
ASV956	0.04a,b	0.03a,b	0.07a	0.03b	0.009	0.950	0.240	0.010
ASV79	0.79a,b	0.55b	0.94a	0.54b	0.118	0.870	0.120	0.010
ASV140	0.14b	1.04a	0.63a,b	0.52a,b	0.287	0.890	0.460	0.010
ASV111	0.65a	0.2b	0.54a	0.49a,b	0.123	0.970	0.340	0.010
ASV532	0.62a	0.07b	0.11a,b	0.05b	0.239	0.600	0.260	0.010

Cereal form (C_{soak}) [2] *:*	Fresh	Fresh	Soak	Soak		*P*-value [5]		
Enzyme (ENZ) [3] *:*	-	+	-	+	SEM [4]	ENZ	C_{soak}	ENZ*C_{soak}
ASV129	0.63a	0.26b	0.48a,b	0.26b	0.124	0.860	0.120	0.010
ASV1093	0.19a	0.14a	0.02b	0.09a	0.032	0.530	0.950	0.010
ASV313	0.05b	0.26a	0.49a	0.22a,b	0.148	0.640	0.680	0.010
ASV273	0.16a,b	0.1b	0.17a,b	0.38a	0.086	0.410	0.570	0.010
ASV1148	0.01b	0.06a	0.06a,b	0.08a	0.022	0.680	0.460	0.010
ASV930	0.04a,b	0.07a,b	0.03b	0.1a	0.021	0.930	0.120	0.010
ASV211	0.13b	0.57a	0.14b	0.25a,b	0.168	0.580	0.120	0.010
ASV1585	0.01b	0.05a	0.05a	0.02a,b	0.012	0.870	0.850	0.010
ASV1376	0.05a	0.06a	0.05a	0.01b	0.013	0.640	0.670	0.010
ASV962	0.06b	0.06b	0.18a	0.04b	0.028	0.410	0.470	0.010
ASV559	0.03b	0.09a,b	0.07a,b	0.14a	0.032	0.410	0.240	0.010
ASV346	0.21a	0.03b	0.18a	0.19a	0.075	0.730	0.630	0.010
ASV16	0.11b	0.73a	0.07b	0.08b	0.293	0.290	0.240	0.010
ASV1599	0.07a	0.12a	0.05a	0.02b	0.014	0.490	0.870	<0.001
ASV20	0.02b	0.02b	0.08a	0.05a,b	0.017	0.000	0.720	<0.001
ASV1364	0.08a	0.06a	0.04a	0.02b	0.004	0.410	0.640	<0.001
ASV437	0.12a,b	0.08b	0.27a	0.08b	0.057	0.600	0.120	<0.001
ASV290	0.09b	0.11b	0.19a,b	0.29a	0.048	0.180	0.500	<0.001
ASV354	0.3a	0.16a,b	0.11b	0.13b	0.043	0.180	0.470	<0.001
ASV926	0.05b	0.1a	0.08a	0.09a	0.007	0.900	0.300	<0.001
ASV209	0.65a	0.15b	0.11b	0.13b	0.194	0.320	0.270	<0.001
ASV1412	0.01b	0.04a	0.04a	0.02a,b	0.005	0.790	0.810	<0.001
ASV908	0.01b	0.1a	0.1a	0.12a	0.014	0.650	0.640	<0.001
ASV1683	0.01 c	0.06b	0.02 c	0.28a	0.039	0.620	0.230	<0.001
ASV320	0.36a	0.05c	0.19a,b	0.1c b	0.108	0.840	0.120	<0.001
ASV957	0.02b	0.07a	0.07a	0.07a	0.014	0.600	0.570	<0.001
ASV2257	0.00a	0.05b	0.01a	0.01a	0.002	0.000	0.470	<0.001
ASV2266	0.03b	0.02 c	0.03a	0.01 d	0.000	0.990	0.320	<0.001
ASV317	0.06b	0.26a	0.13a,b	0.21a	0.044	0.990	0.120	<0.001
ASV562	0.13a	0.06b	0.12a	0.1a	0.009	0.860	0.120	<0.001

[1] Values that do not share a common superscript are statistically different ($P < 0.05$).

[2] Soaking the cereal fraction of the diet prior to feeding (fresh = 0h soaking, soak = 3h soaking).

[3] Enzyme supplementation with a xylanase and β-glucanase complex [unsupplemented (-), supplemented (+)].

[4] SEM = standard error of the mean.

[5] P-value corrected for false discovery rate (FDR).
